# Complete genome sequence of *Enterobacter* sp. IIT-BT 08: A potential microbial strain for high rate hydrogen production

**DOI:** 10.4056/sigs.4348035

**Published:** 2013-12-15

**Authors:** Namita Khanna, Ananta Kumar Ghosh, Marcel Huntemann, Shweta Deshpande, James Han, Amy Chen, Nikos Kyrpides, Kostas Mavrommatis, Ernest Szeto, Victor Markowitz, Natalia Ivanova, Ioanna Pagani, Amrita Pati, Sam Pitluck, Matt Nolan, Tanja Woyke, Hazuki Teshima, Olga Chertkov, Hajnalka Daligault, Karen Davenport, Wei Gu, Christine Munk, Xiaojing Zhang, David Bruce, Chris Detter, Yan Xu, Beverly Quintana, Krista Reitenga, Yulia Kunde, Lance Green, Tracy Erkkila, Cliff Han, Evelyne-Marie Brambilla, Elke Lang, Hans-Peter Klenk, Lynne Goodwin, Patrick Chain, Debabrata Das

**Affiliations:** 1 Indian Institute of Technology Kharagpur, Kharagpur, West Bengal, India; 2 Department of Energy Joint Genome Institute, Walnut Creek CA USA; 3 Lawrence Livermore National Laboratory, Livermore CA USA; 4 Leibniz-Institute—DSMZ – German Collection of Microorganisms and Cell Cultures, Braunschweig, Germany

**Keywords:** *Enterobacter* sp. IIT-BT 08, genome sequence, facultative anaerobe, biohydrogen

## Abstract

*Enterobacter* sp. IIT-BT 08 belongs to Phylum: *Proteobacteria*, Class: *Gammaproteobacteria*, Order: *Enterobacteriales*, Family: *Enterobacteriaceae*. The organism was isolated from the leaves of a local plant near the Kharagpur railway station, Kharagpur, West Bengal, India. It has been extensively studied for fermentative hydrogen production because of its high hydrogen yield. For further enhancement of hydrogen production by strain development, complete genome sequence analysis was carried out. Sequence analysis revealed that the genome was linear, 4.67 Mbp long and had a GC content of 56.01%. The genome properties encode 4,393 protein-coding and 179 RNA genes. Additionally, a putative pathway of hydrogen production was suggested based on the presence of formate hydrogen lyase complex and other related genes identified in the genome. Thus, in the present study we describe the specific properties of the organism and the generation, annotation and analysis of its genome sequence as well as discuss the putative pathway of hydrogen production by this organism.

## Introduction

Hydrogen has great promise in contributing substatially to the renewable energy demands of the future. It is considered a dream fuel by virtue of the fact that it is renewable, does not evolve green house gases, has the highest energy content per unit mass of any known fuel (143 GJ t^-1^), is easily converted to electricity by fuel cells and upon combustion, gives water as the only byproduct [1]. Moreover, hydrogen is the third most abundant element on Earth. However, finding simple, inexpensive ways to extract hydrogen and produce it in a pure gaseous form is a crucial step toward making the "hydrogen economy" a reality. Considering this, hydrogen production using microbes is thought to be a promising technique to produce economical, abundant hydrogen without utilizing fossil fuels. Many microbial species have been reported for hydrogen production [2]. Among them, *Enterobacter* sp. IIT-BT 08 (MTCC 5373, DSM 24603) was reported as a high rate hydrogen producer [3]. It is a Gram negative, facultative anaerobe that can grow and produce hydrogen from a wide range of simple sugars and complex polysaccharides [4]. In the past decade, the group at the Bioprocess Engineering Laboratory at IIT Kharagpur, India, has extensively worked on this organism using various fermentative approaches and established it as one of the highest yielding hydrogen producers [5]. The novelty of the organism lies in the amount of hydrogen (2.2 mol H_2_ mol^-1^ glucose) it can produce at ambient temperature (37 **°**C) and atmospheric pressure as compared to other closely related species reported in literature. Besides, high rate of continuous hydrogen production has been reported using immobilized *Enterobacter* sp. IIT-BT 08 and waste as substrate using 20 L and 800 L reactors [5]. Therefore, whole genome sequencing of this potential strain was considered to determine the genes responsible for the high rate hydrogen production. In this report we present a summary of the properties and features of *Enterobacter* sp. IIT-BT 08 genome and also suggest a putative pathway for hydrogen production.

## Classification and features

**Figure 1:**
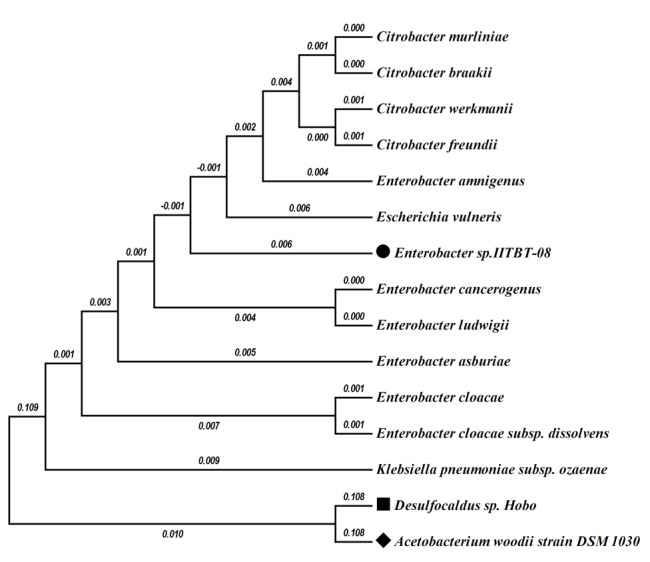
Phylogenetic tree high-lighting the position of *“Enterobacter* sp. IIT-BT 08 (•)” relative to other type and non-type strains within the *Enterobacteriaceae*. Strains shown are those within the *Enterobacteriaceae* having corresponding NCBI genome project ids. The tree was constructed using Mega4 software. The tree based on Jukes–Cantor distance was constructed using neighbor-joining algorithm with 1,000 bootstrapping. *Acetobacterium woodii* strain DSM 1030 (⬥) and *Desulfocaldus* sp. (■) was considered as the out group. The scale bar represents 0.1 substitutions per nucleotide position. Numbers at the nodes are the bootstrap values.

*E. sp.* IIT-BT 08 was isolated from the leaves of a local plant near the Kharagpur railway station, Kharagpur, West Bengal, India [4]. The bacterium is a Gram negative, small, motile, catalase positive rod [4,6,7] belonging to the family *Enterobacteriaceae* (Table 1). To characterize the strain, a set of standard tests were carried out according to Bergey’s Manual and the results showed that the strain belongs to *Enterobacter* species*.* 16S rRNA sequencing by Microbial Type Culture Collection (MTCC), Chandigarh further confirmed the strain identity. The genetic complexity of the organism is illustrated in the phylogenetic tree of the 16S RNA region (Figure 1). Initially the strain was classified as *Enterobacter sp.* IIT-BT 08, however, whole genome sequencing of the strain revealed sequence variation in the six 16S rRNA copies of the strain. We presume that this may have been the source of difficulty in the initial mis-identification of the strain. Currently, without a complete set of type strain genome sequences available for a more detailed taxonomic identification, the name of the strain has been changed to *Enterobacter* sp. IIT-BT 08.

**Table 1:** Classification and general features of *Enterobacter sp.* IIT-BT 08 according to the MIGS recommendation [8]

**MIGS^a^ ID**	**Property**	**Term**	**Evidence code**^b^
		Domain *Bacteria*	TAS [9]
		Phylum *Proteobacteria*	TAS [10]
		Class *Gammaproteobacteria*	TAS [11-13]
	**Current classification**	Order *Enterobacteriales*	TAS [14]
		Family *Enterobacteriaceae*	TAS [15-17]
		Genus *Enterobacter*	TAS [15,17-20]
		Species *Enterobacter* sp.	[15,18,20]
		Type strain IIT-BT 08	
	Gram stain	Negative	NAS
	Cell shape	Rod shaped	IDA
	Motility	motile via peritrichous flagella	IDA
	Sporulation	Non-sporulating	IDA
	Temperature range	25 – 40 °C	TAS [4]
	Optimum temperature	37 °C	TAS [4]
	Carbon source	carbohydrates	TAS [4]
	Energy metabolism	Chemoorganotrophic	IDA
	Terminal electron receptor	Oxygen	IDA
MIGS-6	Habitat	Plant leaves	TAS [4]
MIGS-6.3	Salinity	Not studied	
MIGS-22	Oxygen	Facultative anaerobe; grows well under oxic and anoxic conditions	TAS [4]
MIGS-15	Biotic relationship	Free-living	IDA
MIGS-14	Pathogenicity	None	
MIGS-4	Geographic location	Kharagpur in the district of West Midnapur, West Bengal, India.	TAS [4]
MIGS-5	Sample collection time	July, 1999	
MIGS-4.1	Latitude	22 02’ 30”	
MIGS-4.2	Longitude	87 11’ 0”	
MIGS-4.3	Depth	NA	
MIGS-4.4	Altitude	NA	

a) MIGS: The minimum information about a genome sequence

b) Evidence codes - IDA: Inferred from Direct Assay; TAS: Traceable Author Statement (i.e., a direct report exists in the literature); NAS: Non-traceable Author Statement (i.e., not directly observed for the living, isolated sample, but based on a generally accepted property for the species, or anecdotal evidence). These evidence codes are from the Gene Ontology project [21].

## Genome project history

### Genome sequencing information

*Enterobacter sp.* IIT-BT 08 is a promising hydrogen producer and can utilize waste as substrate for hydrogen production [4]. Therefore, it was considered essential to sequence the whole genome of the organism to determine the genes that contributed towards hydrogen production. Besides, complete genome information was also critical to facilitate studies on genetic engineering of the organism for further enhancement of its hydrogen production potential. Therefore, the group applied for the Community Sequencing Program-2010 (CSP-2010) offered by DoE-JGI.

One of the DOE missions is to address the critical question of depleting energy reserves by creating a new generation of biological research enabled by the genome revolution. This organism therefore appeared relevant to this mission and was selected for sequencing. The genome sequence was completed on May 21, 2012. Quality assurance was done by the DSMZ (Braunschweig, DE), finishing and annotation was completed at Joint Genome Institute. A summary of the project information is shown in Table 2, which also presents the project information and its association with MIGS version 2.0 compliance [8].

**Table 2:** Genome sequencing project information

**MIGS ID**	**Property**	**Term**
MIGS-31	Finishing quality	High-quality draft
MIGS-28	Libraries used	IGHT and IGGH
MIGS-29	Sequencing platforms	Illumina, 454
MIGS-31.2	Fold coverage	50×
MIGS-30	Assemblers	Velvet v. 1.1.05, ALLPATHS v. 39750, Phrap v. 4.24
MIGS-32	Gene calling method	Gene Prodigal
	Genome Database release	September 6^th^, 2012
	NCBI ID	1070842
	Genbank Date of Release	Not determined
	GOLD ID	Gi12106
	Project relevance	Biohydrogen production

### Growth conditions and DNA isolation

For genomic DNA isolation, *Enterobacter* sp. was cultivated overnight in nutrient broth at 37 °C and 200 rpm in a gyratory incubator shaker. DNA isolation was carried out by Deutsche Sammlung von Mikroorganismen und Zellkulturen GmbH (DSMZ) institute. For DNA isolation, the strain was grown in DSMZ medium 381 (Luria-Bertani Medium) at 37°C. DNA was isolated from 1-1.5 g of cell paste using Jetflex Genomic DNA Purification Kit (Genomed_600100) following the manufacturer’s recommendations for Gram-positive bacteria (which were more efficient than the conditions recommended for Gram-negative cells). The identity of the DNA was confirmed via 16S rRNA gene sequencing and the quality was analyzed following the recommendations of the sequencing center (JGI), including pulse-field gel electrophoresis.

### Genome sequencing and assembly

The draft genome of *Enterobacter* sp. IIT-BT 08 was generated at the DOE Joint Genome Institute (JGI) using Illumina data [22]. For this genome, JGI constructed and sequenced an Illumina short-insert paired-end library with an average insert size of 231 +/- 59 bp which generated 24,130,984 reads and an Illumina long-insert paired-end library with an average insert size of 8,267 +/- 2,204 bp which generated 13,553,468 reads totaling 5,653 Mbp of Illumina data. (unpublished, Feng Chen). All general aspects of library construction and sequencing performed at the JGI can be found at the JGI website. The initial draft assembly contained 21 contigs in 3 scaffold(s). The initial draft data was assembled with Allpaths, version 39750, and the consensus was computationally shredded into 10 Kbp overlapping fake reads (shreds). The Illumina draft data was also assembled with Velvet, version 1.1.05 [23], and the consensus sequences were computationally shredded into 1.5 Kbp overlapping fake reads (shreds). The Illumina draft data was assembled again with Velvet using the shreds from the first Velvet assembly to guide the next assembly. The consensus from the second Velvet assembly was shredded into 1.5 Kbp overlapping fake reads. The fake reads from the Allpaths assembly and both Velvet assemblies and a subset of the Illumina CLIP paired-end reads were assembled using parallel phrap, version 4.24 (High Performance Software, LLC). Possible mis-assemblies were corrected with manual editing in Consed [24-26]. Gap closure was accomplished using repeat resolution software (Wei Gu, unpublished), and sequencing of bridging PCR fragments with Sanger and/or PacBio (unpublished, Cliff Han) technologies. For improved high quality draft and noncontiguous finished projects, one round of manual/wet lab finishing may have been completed. Primer walks, shatter libraries, and/or subsequent PCR reads may also be included for a finished project. A total of 0 additional sequencing reactions, 6 PCR PacBio consensus sequences, and 0 shatter libraries were completed to close gaps and to raise the quality of the final sequence. The total estimated size of the genome is 4.7 Mb and the final assembly is based on 5,653 Mbp of Illumina draft data, which provides an average 1,203× coverage of the genome.

### Genome annotation

Genes were identified using Prodigal [27] as part of the Oak Ridge National Laboratory genome annotation pipeline, followed by a round of manual curation using the JGI GenePRIMP pipeline [28]. The predicted CDSs were translated and used to search the National Center for Biotechnology Information (NCBI) non-redundant database, Uni-Prot, TIGRFam, Pfam, PRIAM, KEGG, COG, and InterPro databases. Additional gene prediction analysis and functional annotation were performed within the Integrated Microbial Genomes Expert Review (IMG-ER) platform [29].

## Genome properties

**Figure 2:**
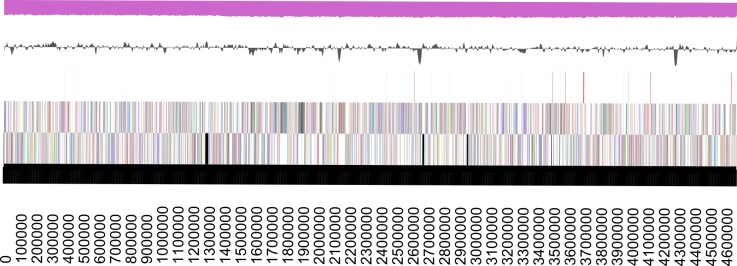
Graphical linear map of the genome. From left to right: Genes on forward strand (color by COG categories), Genes on reverse strand (color by COG categories), RNA genes (tRNAs green, rRNAs red, other RNAs black), GC content, GC skew.

The genome of *E. sp.* IIT-BT 08 consists of one linear chromosome of 4,672,040 bp (Figure 2). The average G+C content for the genome is 56.01% (Table 3). There are 78 tRNA genes and 6 rRNA operons each consisting of a 16S, 23S, and 5S rRNA gene. There are 4,393 predicted protein-coding regions and 43 pseudogenes in the genome. A total of 3,881 protein-coding genes (85.64%) have been assigned a predicted function while the rest have been designated as hypothetical proteins (Table 4). The numbers of genes assigned to each COG functional category are listed in Table 4. About 2% of the annotated genes were not assigned to COGs and have an unknown function.

**Table 3:** Nucleotide content and gene count levels of the genome

**Attribute**	Value	% of total^a^
Genome size (bp)	4,672,040	100.00
DNA coding region (bp)	4220,082	90.33
DNA G+C content (bp)	2,616273	56.01
Total genes	4,532	100.00
RNA genes	179	3.95
Protein-coding genes	4,393	96.05
Genes in paralog clusters	1,665	36.74
Genes assigned to COGs	3,780	83.41
Genes assigned Pfam domains	3,949	87.14
Genes assigned TIGRfam domains	1,715	37.84
Genes with signal peptides	1,603	35.37
Genes with transmembrane helices	1,101	24.29
Pseudo Genes^b^	43	0.95

a) The total is based on either the size of the genome in base pairs or the total number of protein coding genes in the annotated genome.

b) Pseudogenes may also be counted as protein coding or RNA genes, so is not additive under total gene count.

**Table 4:** Number of genes associated with the general COG functional categories

**Code**	**Value**	**%age**^a^	**Description**
J	191	4.4	Translation
A	1	0.0	RNA processing and modification
K	372	8.5	Transcription
L	153	3.5	Replication, recombination and repair
B	0	0.0	Chromatin structure and dynamics
D	32	0.7	Cell cycle control, mitosis and meiosis
Y	0	0.0	Nuclear structure
V	56	1.3	Defense mechanisms
T	220	5.1	Signal transduction mechanisms
M	249	5.7	Cell wall/membrane biogenesis
N	148	3.4	Cell motility
Z	0	0.0	Cytoskeleton
W	0	0.0	Extracellular structures
U	155	3.6	Intracellular trafficking and secretion
O	146	3.4	Posttranslational modification, protein turnover, chaperones
C	244	5.6	Energy production and conversion
G	396	9.1	Carbohydrate transport and metabolism
E	402	9.2	Amino acid transport and metabolism
F	83	1.9	Nucleotide transport and metabolism
H	165	3.8	Coenzyme transport and metabolism
I	105	2.4	Lipid transport and metabolism
P	246	5.7	Inorganic ion transport and metabolism
Q	80	1.8	Secondary metabolites biosynthesis, transport and catabolism
R	446	10.2	General function prediction only
S	375	8.6	Function unknown
-	88	2.0	Not in COGs

^a^The total is based on the total number of protein coding genes in the entire annotated genome

### Biohydrogen production pathway

The complete genome sequencing of the organism helps provide a preliminary idea of the genes involved in the hydrogen production pathway. The genome revealed the presence of formate hydrogen lyase (EntIIITBT8_2511) and its maturation operons HycH (EntIIITBT8_2678), NiFe hydrogenase III small and large subunit (EntIIITBT8_2679, EntIIITBT8_2681), their maturation operons and the FeS cluster containing hydrogenase components 1 and 2 (EntIIITBT8_0331, EntIIITBT8_2684). A complete list of all the genes predicted to be involved in the hydrogen production pathway is listed in Table 5. The whole genome information of the organism suggests that hydrogen production in *Enterobacter* sp. IIT-BT 08 is carried out through the formate hydrogen lyase (FHL) complex which consists of formate dehydrogenase (FDH-H), hydrogenase (Hyd-3) and the electron transfer mediators [30].

**Table 5:** Preliminary genes involved in the hydrogen production pathway according to the MIGS recommendations [8]

**Protein product**	**Gene locus**	**Molecular mass (kDa)**	**Description**
FdhD	EntIITBT8DRAFT_3548	29.9	formate dehydrogenase family accessory protein (protease)
FdhE	EntIITBT8DRAFT_3551	89.4	formate dehydrogenase, alpha subunit, proteobacterial-type [EC:1.2.1.2 ]
FdhF	EntIITBT8DRAFT_1016	79.3	formate dehydrogenase, alpha subunit, archaeal-type (EC:1.2.1.2)
HycA	EntIITBT8DRAFT_2685	17.5	Transcriptional repressor of hyc and hyp operons
HycD	EntIITBT8DRAFT_2682	33.0	Formate hydrogenlyase subunit 4
HycE	EntIITBT8DRAFT_2681	64.8	Ni, Fe-hydrogenase III large subunit
HycG	EntIITBT8DRAFT_2679	28.0	Ni,Fe-hydrogenase III small subunit
HycH	EntIITBT8DRAFT_2678	15.2	Formate hydrogenlyase maturation protein
HycI	EntIITBT8DRAFT_2677	16.2	hydrogenase maturation protease [EC:3.4.23.51]
?	EntIITBT8DRAFT_2511 EntIITBT8DRAFT_2680	23.0	Formate hydrogenlyase subunit 6/NADH:ubiquinone oxidoreductase subunit (chain I)
?	EntIITBT8DRAFT_2683	63.5	Formate hydrogenlyase subunit 3/Multisubunit Na+/H+ antiporter, MnhD subunit
HycB??	EntIITBT8DRAFT_2684	21.7	Fe-S-cluster-containing hydrogenase components 2
			
HypA	EntIITBT8DRAFT_2686	13.1	hydrogenase nickel insertion protein
HypB	EntIITBT8DRAFT_2687	31.1	hydrogenase accessory protein
HypC/HupF	EntIITBT8DRAFT_2688	9.65	hydrogenase assembly chaperone HypC/HupF
HypD	EntIITBT8DRAFT_2689	41.1	hydrogenase expression/formation protein HypD
HypE	EntIITBT8DRAFT_2690	35.3	hydrogenase expression/formation protein HypE
HypF	EntIITBT8DRAFT_2672	80.5	[NiFe] hydrogenase maturation protein HypF
HoxN/HupN/NixA family	EntIITBT8DRAFT_2671	36.7	high-affinity nickel-transporter

**Figure 3:**
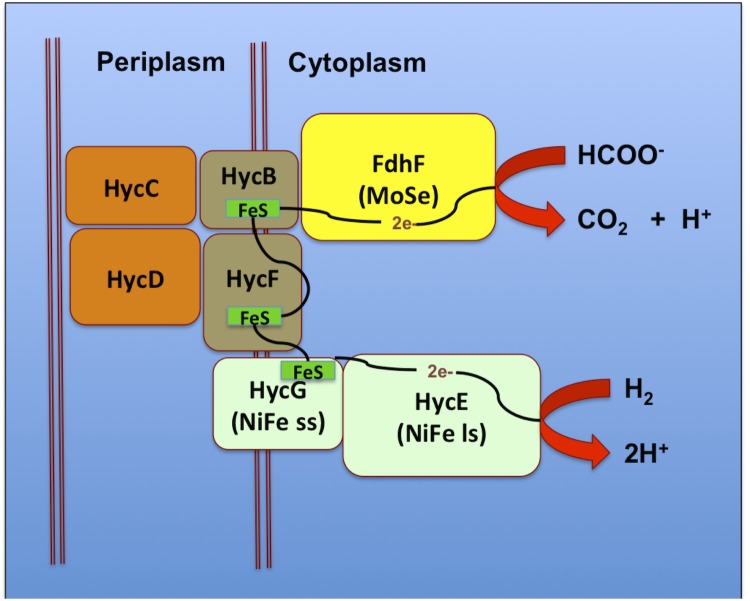
Putative mechanism of hydrogen production by *Enterobacter* sp. IIT-BT 08 based on the genes identified in the genome. Figure is adapted from [33].

However, in the future the hypothetical pathway must be verified with wet lab experiments. Based on the previous reported literature it may be that formate dehydrogenase and hydrogenase 3 together form a membrane protein complex that is responsible for hydrogen production in facultative anaerobes [30-32]. Rossmann et al. suggested that in facultative anaerobes hydrogen production was determined by the concentration of formate in the cell, which in turn determined the formation of the FHL complex [32]. A putative model (Figure 3) has been suggested based on the biochemistry of the reactions involved in the pathway [34]. Formate dehydrogenase is suggested to catalyze the oxidation of formate into carbon dioxide. The electrons released in the process are transferred to Hyd3 encoded by hycABCDEFGH to generate molecular hydrogen under anaerobic conditions [33]. The model suggests a plausible scheme of electron transfer from FdhF to the catalytic subunit of hycE via hycBCFG subunits. Among these, hycB and hycF have been determined to be [4Fe-4S] ferredoxin type electron transfer proteins [35]. On the other hand, hycE and hycG shares homology with NADH ubiquinone oxidoreductase (NUO) subunits of the mitochondria and chloroplast [35]. In the model, hycC and hycD have been suggested to act as transmembrane proteins.

Electron acceptors, like oxygen or nitrate, generally inhibit the expression of the FHL complex, whereas its biosynthesis is controlled by the concentration of formate in the cell [32]. Further, it has been suggested that the micro elements selenium and molybdenum are involved at the active site of FDH-H, while nickel is a component of the Hyd-3 active site [30,36]. Accordingly, it has been suggested that the FHL complex can be induced by regulating the presence of formate and metal ions in slightly acidic pH under anaerobic conditions.

Transcription of the FHL complex is under the control of several genes, including *fhlA*, which codes for the FHL activator protein FHLA, a tetramer that binds to the upstream region of the DNA encoding the FHL complex and promotes the transcription of the FHL complex [34,37]. Moreover, *hycA* codes for the FHL repressor protein that binds to FHLA or to the FHLA-formate complex. Since *fhlA* and *hycA* control the transcription of the FHL complex, it is theoretically possible to control the specific FHL activity and the specific hydrogen production rate by manipulating these genes or their genetic controls [38].

## Conclusion

The genome of *Enterobacter sp.* IIT-BT 08 was sequenced and annotated by the DOE Joint Genome Institute. The genomic properties of the organism were analyzed using various IMG tools, and, based on the genome sequence, a putative pathway of hydrogen production based on formate hydrogen lyase complex was discussed.
